# Allergen immunotherapy for food allergy: Evidence and outlook 

**DOI:** 10.5414/ALX02319E

**Published:** 2022-11-21

**Authors:** Antonella Muraro, Angelo Tropeano, Mattia Giovannini

**Affiliations:** 1 Food Allergy Referral Centre Department of Mother and Child Health, University of Padua, Padua,; 2 Department of Human Pathology of Childhood and Adulthood, University of Messina, Messina, and; 3 Allergy Unit, Department of Pediatrics, Meyer Children’s University Hospital, Florence, Italy

**Keywords:** allergen immunotherapy, food allergy, OIT, SLIT, EPIT

## Abstract

Food allergy represents a significant health issue characterized by a sizeable epidemiological burden, involving up to 5% of adults and up to 8% of children in the Western world. The elimination diet of the trigger food is the cornerstone of food allergy management. However, novel treatment options are most wanted to provide alternative strategies for this potentially fatal medical condition. Allergen immunotherapy for food allergy (FA-AIT) is considered an immunomodulatory intervention where regular exposure to increasing doses of food is performed in the context of an allergist’s supervised protocol. The main objective is to decrease reactivity, attenuate life-threatening allergic episodes and reduce frequent access to the emergency department. Achieving food tolerance off-treatment is, however, the ultimate aim. In this review, we aim to summarize FA-AIT evidence and outlook.

## Key messages

- Different routes of food administration have been investigated for allergen immunotherapy for food allergy (FA-AIT): oral, sublingual, epicutaneous, and subcutaneous.
- There is currently evidence that oral immunotherapy (OIT) is effective for peanut, milk, and egg and epicutaneous immunotherapy (EPIT) for peanut compared to sublingual immunotherapy (SLIT) and subcutaneous immunotherapy (SCIT); however, a higher frequency of adverse events is reported for OIT compared to EPIT.
- Several unmet needs should be investigated in the coming years to optimize the role of FA-AIT in the treatment of IgE-mediated food allergy including the role of biologics.

## Introduction 

**Figure 1:**
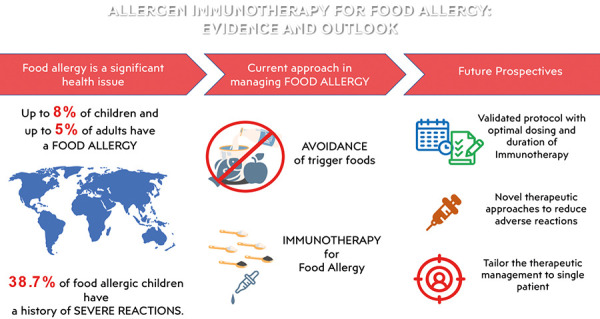
Allergen immunotherapy for food allergy: evidence and outlook.

Food allergy represents a significant health issue characterized by a sizeable epidemiological burden, involving up to 5% of adults and up to 8% of children in the Western world [1, 2]. Thus, even if the elimination diet of the trigger food is the cornerstone of food allergy management [3, 4], novel treatment options are most wanted to provide alternative strategies for this potentially lethal medical condition (Figure 1). 

Allergen immunotherapy for food allergy (FA-AIT) is intended as an immunomodulatory intervention for IgE-mediated food allergy based on recurrent exposure to increasing doses of food at regular intervals. This process of desensitization had been set up with the main initial objective to attenuate severe allergic manifestations and reduce frequent access to the emergency department. Desensitization is intended to increase the patient’s threshold required to elicit an allergic reaction, consequently reducing the risks related to accidental food ingestion, and it is linked to regular food exposure. Should the allergen administration be interrupted, the previous level of clinical reactivity may return. This practice was reported for the first time in 1908 for hen’s egg allergy [5]; however, since then, several routes of food allergen administration have been investigated (e.g., oral, sublingual, epicutaneous, and subcutaneous). It is apparent that the different amount of allergen utilized, depending on the administration route, is associated with different effectiveness and rate of adverse events [6] (Table 1). 

The European Academy of Allergy and Clinical Immunology (EAACI) has provided guidelines to support interested clinicians in the best use of FA-AIT [2] based on a formal systematic review and meta-analysis of the evidence in the field [7]. A systematic review on FA-AIT by the Global Allergy and Asthma European Network (GA2LEN) ANACare group has been published, confirming that desensitization is an attainable goal of FA-AIT [8]. There is, however, a potential role of FA-AIT in achieving post-discontinuation effectiveness (also known as tolerance or sustained unresponsiveness). It refers to the absence of clinical manifestations after ingestion of a regular serving of the culprit food after a prolonged period without administration of the active treatment. In this review, we aim to summarize FA-AIT evidence and outlook. 

## Oral immunotherapy 

### Basic aspects and general indications 

Oral immunotherapy (OIT) involves administering increasing doses of an allergenic food via the oral route with the aim to increase the threshold of reaction in patients with persistent food allergies [6, 9]. However, the ability of OIT to induce sustained tolerance when the treatment is stopped seems limited at present. It appears to be more probable when OIT is continued for a long time, as demonstrated for egg-allergic patients [10], and when it starts at a younger age, as evidenced in peanut-allergic children [11]. 

Currently, OIT is recommended for persistent cow’s milk, hen’s egg, and peanut allergy [12]. 

Considering that many children with allergies to cow’s milk or hen’s egg develop tolerance spontaneously at preschool age, it is reasonable to propose OIT to children from ~ 4 – 5 years of age [2, 12]. So far, OIT has been performed using essentially fresh material or native foods. This may impair the allergen content. Moreover, there are differences in the form of food and administration schedules used for desensitization. Indeed, a newly licensed product is now available exclusively for peanut OIT, which paves the way for opportunities to have standardized products prepared according to Good Manufacturer Practice (GMP) and approved by regulatory authorities. 

### Protocols 

As a general rule, before starting FA-AIT, an oral food challenge is necessary to establish the threshold of reaction to the culprit food. The usual schedule entails a build-up phase, in which small amounts of food are given to the patient in a hospital setting, and then the highest tolerated dose is administered at home daily. The doses are increased usually at regular intervals. e.g., weekly, at the outpatient clinic until reaching a maintenance dose that the patient has to take daily for the entire length of the schedule. 

Currently, there is no international validated protocol with optimal dosing and duration of therapy, and different schedules have been used for clinical trials (e.g., rush immunotherapy, slow up-dosing regimen, and weekly schedule). 

### OIT for cow’s milk allergy 

For cow’s milk allergy, liquid pasteurized raw milk is most often preferred. However, considering that up to 75% of children with cow’s milk allergy tolerate baked-milk products (e.g., muffin, waffles) or heated milk [13, 14] and that regular exposure to baked-milk products significantly accelerates the development of unheated-milk tolerance [15], different protocols with baked-milk products or heated milk have been used in the last years. A study was performed on children with severe cow’s milk allergy comparing unheated versus heated milk. The treatment efficacy was 50%, but the incidence of adverse events was high, suggesting that standardization of OIT with cow’s milk requires further investigation, with priority placed on safety. Additional data on the long-term effect of OIT with cow’s milk were provided, showing that the effect of OIT had a reasonably long persistence [14]. 

If the desensitization to fresh milk was designed as the primary outcome, the rate of success of baked-milk OIT varies between 9 and 88.1% [13, 15], 16]. This wide range essentially depends on protocols used and inclusion criteria selected, i.e., the inclusion of patients with a more severe allergic phenotype (e.g., history of systemic manifestations, elevated specific IgE levels, atopic comorbidities). Nevertheless, a desensitization rate of 42.2% was reported in children suffering from anaphylactic reactions after baked-milk OIT [16]. A phase II randomized, double-blind, placebo-controlled study compared the safety and efficacy of baked-milk OIT versus placebo in highly allergic children with cow’s milk allergy. After 12 months of treatment, 73% of children in the baked-products arm tolerated 4,044 mg of baked-milk protein compared with 0% of children in the placebo arm. Dose-related reactions, although common, were mild in the vast majority of cases [17]. 

### OIT for egg allergy 

For egg allergy, OIT can be performed with pasteurized raw egg white, extensively heated egg (e.g., omelette and/or boiled egg white and egg yolk), dehydrated egg white in powder or baked egg products. It is worth noting that the time of cooking has a greater effect on egg allergenicity than the temperature used [18] and that ovomucoid, the dominant allergen in egg white, is a heat-stable protein. Although some cohort studies have suggested that the consumption of baked eggs quickly results in immune changes and tolerance acquisition to raw egg [19, 20, 21], others did not confirm these data [22, 23]. 

### OIT for peanut allergy 

OIT with peanut can be performed with defatted peanut flour [24, 25], crushed roasted peanuts [26], or boiled peanuts [27]. It should be highlighted that for peanut allergy, dry roasting augments allergenic potential in contrast to boiling and frying. Indeed, the Maillard reaction induces the formation of protein aggregates that are more resistant to gastric digestion and that bind IgE antibodies more effectively [28]. As mentioned above, due to high-quality data on this specific area [11], a drug recently licensed approved by the European Medicines Agency (EMA) and the Food and Drug Administration (FDA) is available only for peanut-allergic children and adolescents aged from 4 to 17 years. This product is a defatted peanut powder containing capsules and sachets, sprinkled into a vehicle (e.g., ice cream or applesauce) and consumed daily. All up-dosing should occur under medical supervision, whereas the patient will continue daily dosing at home; on reaching the maintenance dose, daily dosing should continue at home indefinitely. 

### OIT effectiveness 

The success rates of OIT vary from 36 to 90% depending on the considered food allergen as well as the outcomes in the different trials. Albeit many questions remain still unsolved (e.g., optimal maintenance dose, duration of OIT, reliable biomarkers that predict favorable outcome), OIT has the largest body of evidence among FA-AIT and results more efficacious than sublingual immunotherapy (SLIT) [7, 12] 

If compared with SLIT, the typical doses administered during OIT are more abundant (in order of milligrams versus a few micrograms in SLIT); consequently, adverse events are more frequent in OIT than in SLIT. 

### OIT safety 

Almost all patients experience adverse events during the initial phases of the schedule with a subsequent reduction during the maintenance phase. These are mainly mild (e.g., itching of the oropharynx, perioral rash, mild abdominal pain) and resolve spontaneously or with oral antihistamines; additionally, they may occur with a specific temporal latency from the dose administration. Rarely, adverse events may evolve into more severe systemic reactions, and only a minority of patients experience these [2, 29]. Gastrointestinal clinical manifestations (e.g., nausea, vomiting, abdominal pain, reflux) are the main reason for OIT discontinuation, although they decrease over time. However, ~ 2.7% of the patients develop eosinophilic esophagitis (EoE) after OIT. Although it is still debated whether EoE may be a possible secondary long-term effect of OIT, it is recommended to monitor patients for signs and symptoms of new-onset EoE [2]. 

## Sublingual immunotherapy 

SLIT involves administering increasing doses of an allergenic food via the sublingual route [6]. Data on SLIT effectiveness and safety in the literature are scarce. 

A randomized, double-blind, placebo-controlled trial on SLIT for peanut allergy has been carried out, also including adolescent patients. After the treatment, 70% of subjects were responders, compared to 15% of subjects who received placebo. Moreover, 63.1% of the peanut doses were free of adverse clinical manifestations, 95.2% excluding oral-pharyngeal clinical manifestations. The level of induced desensitization was modest, interestingly with significant increases in the successfully consumed dose with a longer duration of the therapy [30]. 

A systematic review and meta-analysis published on the topic demonstrated relevant benefits in terms of desensitization [7], even if overall SLIT results are less effective than OIT [2]. On the other side, as mentioned above, the typical doses administered during SLIT are smaller; consequently, adverse events are less frequent in SLIT than in OIT, with mild systemic reactions. Moreover, in the latter systematic review and metanalysis, systemic reactions in SLIT-treated patients seemed not to diverge from those recorded in the placebo-treated patients [2, 7]. 

## Epicutaneous immunotherapy 

Epicutaneous immunotherapy (EPIT) involves administering a food allergen via an absorbed patch to deliver the food allergen to the skin [6]. Treatment of allergic conditions through allergen administration via the skin has already been taken into consideration in the past century [31]. However, it has been studied in depth in experimental models and clinical trials in the last years, including patches containing an allergen deposit. 

A double-blind, randomized, placebo-controlled trial on EPIT for the treatment of peanut allergy has been carried out in children and young adults. The response rate was 12, 46, and 48% in the placebo, first treatment group (100-μg patch), and second treatment group (250-μg patch) [32], respectively. Adverse reactions recorded were mainly mild and patch-site reactions, in 14.4% of placebo doses and 79.8% of active doses (100-µg and 250-μg patches) [33]. 

Another randomized, double-blind, placebo-controlled trial on EPIT for peanut allergy has been carried out in children. The responder percentage was 35.3% in the active group and 13.6% in the placebo group. Despite a substantial difference (21.7%) between the groups, this did not meet the prespecified criterion for a positive trial result. However, the clinical relevance of not meeting the latter criterion remained to be determined by the authors. Treatment-emergent adverse events were mainly patch application site reactions, and they manifested in a similar percentage in the active group compared to the placebo group, namely 95.4% and 89%, respectively [34]. 

A systematic review and meta-analysis has been published on the topic, comparing EPIT with placebo for peanut and cow’s milk [35]. A relevant efficacy has been shown for peanut EPIT and, though less strong, for cow’s milk EPIT. EPIT appeared not to increase systemic adverse events, and serious adverse events were similar in the active and placebo groups. On the other side, an increase in local treatment-related adverse events has been outlined. 

EPIT for peanut allergy appears associated to a lower frequency of adverse events than OIT. 

## Future perspectives 

Evidence on FA-AIT is steadily growing [8]. Studies in the literature are often heterogeneous, including the outcomes analyzed for effectiveness and safety [2]. Future efforts should be carried out in setting homogeneous outcomes measures to allow comparison among the studies as far as administration routes, length of the treatment, and sustained unresponsiveness are concerned. Protocols used for FA-AIT should be validated with optimal dosing of the food and duration of the treatment. However, this is particularly difficult to obtain, as the intervention is food-specific and carried out in different clinical and research environments in countries with different eating habits. Adequate standardization of the food products used for AIT is a crucial component of the process with fixed, reproducible quality, ensuring consistency among allergen content, biological potency, lack of contaminants, and overall safety. In this regard, licensed products prepared under GMP for pharmaceutical products and approved by independent governmental bodies are hugely required [36]. In addition, it has been shown in some studies that poor quality of life is the determining factor for a patient to decide for peanut OIT. Thus, patient-related outcomes, such as quality of life and cost-effectiveness, should always be included in any FA-AIT trial and with comparison among different AIT routes [2]. Multidisciplinary working groups, including patients and their representatives, should discuss these outcomes, which should be both significant for the physicians/researchers and people affected by the medical condition. Benefits should outweigh costs. Every treatment should be ideally sustainable by the patients or by the community, with at least partial reimbursement by insurance or by the national health system. 

Basic scientists should commit to advancing our understanding of the pathophysiological mechanisms of allergic inflammation and other pathways involved in food allergy [37]. This knowledge would achieve new biomarkers to assess specific characteristics of the patients, which may be associated with successful immunomodulation before or during the treatment. Indeed, in a personalized medicine perspective characterized by a tailored approach, the definition of the adequate candidate for FA-AIT, is critical. Many variables have been taken into consideration in several administration routes, e.g., skin prick tests, serum specific IgE, IgG, and IgG4 levels, basophil activation test, cytokines (including IL-4, IL-5, IL-10, and IFN-γ), or B and T cells (including regulatory ones) [38]. In addition, biomarkers may be potential targets for other immunomodulatory therapies as well, e.g., probiotics or biologic drugs. Concerning this latter point, future research should focus on the role of FA-AIT in patients undergoing treatments with biologic drugs or probiotics, alone or in combination with food immunotherapy, as evidence from the studies in the literature are still scarce, especially in patients with multiple food allergies. A systematic review on FA-AIT by the GA2LEN ANACare group has been published confirming that there were too few trials of biologic drugs alone or with FA-AIT to draw conclusions [8, 12]. 

Studies regarding subcutaneous FA-AIT are limited in the literature due to its unfavorable safety profile, characterized by many severe reactions, including anaphylaxis [39, 40]. On the other side, hypoallergenic products based on modified recombinant proteins are under evaluation for potential clinical use, and they may represent a promising future therapeutic alternative [41]. Furthermore, a comprehensive knowledge concerning adjuvants may be a helpful resource in the area, with the aim to optimize the immune response of the products used. 

Recently, the safety and tolerability of a product consisting of several synthetic peptides selected from peanut proteins was assessed in a randomized, double-blind, placebo-controlled phase I trial in peanut-allergic adults [42]. The favorable safety profile of the treatment was confirmed by a lack of basophil activation to the product for intradermal injection and documenting mild or moderate adverse events, the majority of which were transient injection site reactions. 

Another emerging therapy is currently undergoing phase I trials for safety in peanut-allergic patients [43]. This novel compound is a single multivalent peanut (Ara h 1, h 2, h 3) lysosomal associated membrane protein DNA plasmid vaccine, administrated via intradermal injection. Furthermore, mRNA vaccines encoding allergens may represent another promising horizon in the area [44]. 

## Conclusion 

Potentially life-threatening reactions caused by accidental exposure or too restrictive diets may hugely impair the quality of life and the nutritional status of food-allergic patients, especially children. According to recent evidence, FA-AIT may change this landscape with very positive outcomes [8, 12]. However, when proposing FA-AIT, a shared decision-making process should be implemented, taking into consideration, e.g., the patient’s clinical reactivity, probability of persistent severe food allergy, psychosocial and logistic circumstances, including access to standardized, licensed products. The clinician and the center should have specific expertise in managing FA-AIT or receive the appropriate training in this regard. Protocols should be in place, including the execution of oral food challenges, management of potentially severe allergic reactions, such as anaphylaxis, possible rapid access to local intensive care as well as performing dose escalation and monitoring of patients undergoing FA-AIT with a fast track for contacting their physicians. 

Several unmet needs should be investigated in the coming years to optimize the role of FA-AIT for the treatment of IgE-mediated food allergy. Indeed, extensive data from a network of Centers of Excellence sharing best practices and working in a harmonized approach in multicentric clinical trials are eagerly awaited in adults and children [45]. Stakeholders should prioritize the primary investments and define a shared research agenda regarding FA-AIT implementation. This would ultimately optimize tailoring AIT, i.e., “the right FA-AIT to the right patient” utilizing it effectively and safely in daily clinical practice 

## Funding 

None. 

## Conflict of interest 

AM has received speaker’s fee from Aimmune, DVB Technologies, Nestlè Health Sciences, Nutricia, Viatris and is a member of Advisory Board for Viatris, Novartis, Aimmune, Regeneron, Sanofi. AT none. MG none. 

**Table 1.** Characteristics of types of allergen immunotherapy for food allergy (EPIT, SLIT, OIT). Modified from [6]. Most data from the literature come from pediatric clinical trials and are based on peanut OIT.

	EPIT	SLIT	OIT
Allergen dose	+	++	+++
Effectiveness	+ +	++	+++
Adverse reactions	+	++	+++

EPIT = epicutaneous immunotherapy; SLIT = sublingual immunotherapy; OIT = oral immunotherapy.
